# The Cross-Modal Suppressive Role of Visual Context on Speech Intelligibility: An ERP Study

**DOI:** 10.3390/brainsci10110810

**Published:** 2020-11-02

**Authors:** Stanley Shen, Jess R. Kerlin, Heather Bortfeld, Antoine J. Shahin

**Affiliations:** 1 Center for Mind and Brain, University of California Davis, Davis, CA 95618, USA; sshen@ucdavis.edu (S.S.); jrkerlin@ucdavis.edu (J.R.K.); 2 Department of Psychology, University of California Merced, Merced, CA 95343, USA; hbortfeld@ucmerced.edu; 3 Department of Cognitive and Information Sciences, University of California Merced, 5200 N Lake Rd, Merced, CA 95343, USA

**Keywords:** audiovisual integration, word identification, speech intelligibility, cross-modal suppression, auditory evoked potentials

## Abstract

The efficacy of audiovisual (AV) integration is reflected in the degree of cross-modal suppression of the auditory event-related potentials (ERPs, P1-N1-P2), while stronger semantic encoding is reflected in enhanced late ERP negativities (e.g., N450). We hypothesized that increasing visual stimulus reliability should lead to more robust AV-integration and enhanced semantic prediction, reflected in suppression of auditory ERPs and enhanced N450, respectively. EEG was acquired while individuals watched and listened to *clear* and *blurred* videos of a speaker uttering *intact* or highly-intelligible degraded (*vocoded*) words and made binary judgments about word meaning (animate or inanimate). We found that *intact* speech evoked larger negativity between 280–527-ms than *vocoded* speech, suggestive of more robust semantic prediction for the *intact* signal. For visual reliability, we found that greater cross-modal ERP suppression occurred for *clear* than *blurred* videos prior to sound onset and for the P2 ERP. Additionally, the later semantic-related negativity tended to be larger for *clear* than *blurred* videos. These results suggest that the cross-modal effect is largely confined to suppression of early auditory networks with weak effect on networks associated with semantic prediction. However, the semantic-related visual effect on the late negativity may have been tempered by the vocoded signal’s high-reliability.

## 1. Introduction

Looking at a talker’s mouth during conversations can boost comprehension of the speech signal, especially in noisy situations [1,2]. Past work has shown that this effect is partly accomplished via cross-modal enhancement of speech envelope tracking by the auditory cortex (AC) [3], a process that is especially useful in cluttered speech environments. This process mirrors the effect of selective attention, whereby speech envelope tracking in the AC of the attended stream is strengthened [4,5]. However, cross-modal influence on audition is not limited to the speech envelope. The McGurk illusion [6] is a prime example of how visual context alters phonemic representations. We recently showed that the N1 auditory evoked potential (AEP) shifts in amplitude to reflect the N1 of the visually conveyed phoneme as opposed to the auditory conveyed phoneme [7,8].

A consistent finding in audiovisual (AV) studies, is the cross-modal suppressive effect on AEPs [9,10,11,12,13]. Using the consonant vowels (CVs)/ka/,/ta/, and/pa/, van Wassenhove et al. [11] showed that the N1-P2 AEPs were significantly reduced in latency and amplitude for AV versus auditory-only (A-only) stimuli. These researchers concluded that preceding visual context not only speeds up auditory processing, but also streamlines auditory perception by inhibiting redundant auditory representations. That is, since some speech information has already been processed by the often-preceding (and predictive) visual modality, redundant auditory representations are suppressed. In support, a recent intracranial recording study by Karas et al. [14] found that the visually mediated suppressive effect in non-primary AC occurs for AV speech stimuli with visemes that typically precede the phonemes, but not for speech stimuli with visemes that typically follow the phonemes. We should note that the N1 and P2 AEPs are observed frontocentrally at the scalp and occur around 100 ms (N1) and 180 (P2) ms following sound onsets. Their main sources are in the primary AC [15], but contributions from non-primary areas cannot be discounted.

The cross-modal AEP suppressive effect has been largely identified in tasks that required perceptual decisions such as AV synchrony or congruency judgments [9,11,12,13,16,17,18]. In a study utilizing AV synchrony judgment of *vocoded* nonwords (see Methods for explanation about vocoding), Shatzer et al. [13] showed that as stimulus reliability increases (i.e., from *blurred* to *clear* or *4*- to *16-channel* vocoding), the P2 AEP reduces in amplitude. However, it was not optimally evident from the Shatzer et al. study how this AEP cross-modal effect is influenced when the task is linguistic (e.g., identify word meaning) as opposed to perceptual (synchrony judgments) in nature. We know that increasing stimulus reliability of the video or audio enhances word intelligibility and the window of AV integration [19]. A semantic task will also evoke linguistically related event related potentials (ERPs) such as the N400 wave—associated with semantic violation [20,21] and evaluation [22]. For example, Kaganovich et al., [21] showed that semantic incongruency between the auditory and visual modalities lead to a greater N400 effect. However, Shahin and Picton [22] showed that semantic evaluation of words evokes larger frontocentral (e.g., Fz and Cz channels) negativities peaking around 450 and 600 ms (N450 and N600) than voice evaluation of the same words. Thus, a linguistic task will evoke additional language-related ERP components over and above a perceptual task for the same stimuli.

In the current study, we evaluated the cross-modal AEP suppressive effect as a function of stimulus reliability while subjects made semantic judgments. Subjects watched and listened to *clear* and *blurred* videos combined with *intact* and noise *vocoded* (24-channel, see Methods for detail) words with either animate or inanimate meanings. The 24-channel vocoding allows for a high degree of intelligibility, thus balancing listening effort between the *vocoded* and *intact* speech as closely as possible, while maintaining some level of phonetic and, in turn, lexical variation. To make sure that participants were attending to both the visual and auditory stimuli, they were required to make judgments on oddball trials of whether a word represented an animate or inanimate object or whether a green oval appeared around the talker’s mouth. If, indeed, the cross-modal suppressive effect reflects the robustness of AV integration, then we should observe suppression of AEPs (P1, N1, and P2) for *clear* versus *blurred* videos, as well as greater enhancement of N450/N600 ERPs, with the latter effect signifying more robust semantic encoding associated with enhanced visual stimulus reliability. We should also observe enhancement of the N450/N600 ERP waves for *intact* versus *vocoded* stimuli for the same abovementioned reasons.

## 2. Materials and Methods

### 2.1. Participants

Twenty-seven participants completed the study. Two participants were excluded from the final analysis because they did not satisfy specific criteria assuring task vigilance: one person acknowledged that they avoided looking at the blurred videos, while another did not respond to 45% of the probe trials (see below). Of the 25 remaining participants, demographic information for two subjects was lost/destroyed and thus not available. Demographic data are thus based on 23 participants (10 males and 13 females, all right-handed). Despite the loss of demographic information on two participants, behavioral and EEG data are available for all 25. Participants (*n* = 23) were English-speaking adults [age: 22.1 ± 4.9 (mean ± SD)] with no known hearing problems or language deficits. They were recruited from the UC Davis campus or local community and tested in the UC Davis Center for Mind and Brain. Participants received monetary remuneration in exchange for completing the study. Informed consent was obtained in accordance with protocols reviewed and approved by the UC Davis Institutional Review Board (IRB) on 26 March 2020 (IRB #725055-1). All procedures followed the approved guidelines of the IRB.

### 2.2. Stimuli

Speech materials included forty monosyllabic animate (e.g., cat) and forty monosyllabic inanimate words (e.g., desk). See Supplementary Table S1 for the full list of words used. All words were highly recognizable, with no ambiguity in terms of animacy. Video and audio were recorded simultaneously from an adult male, using a Panasonic AGDVX100A digital camera at a temporal resolution of 30 fps, and a Shure KSM studio microphone with sampling rate of 48 kHz. The presenter gave informed consent prior to recording in accordance with the ethical guidelines of the University of California. Each audiovisual (AV) word was trimmed into a short video clip about 2.5 s long using Corel VideoStudio Pro X7 (www.videostudiopro.com), such that each clip had about 10 silent frames before and after the speech utterance. Audio and silent video stimuli were extracted and saved separately for subsequent manipulation.

### 2.3. Preparation of Audio Stimuli

Audio stimuli were first converted to mono sound. The audio stimulus preparation produced audio stimuli at two reliability levels: *intact* and *vocoded*. Vocoding was based on the general procedures outlined in Shannon et al. [23]. We used 24-channel vocoding to maintain high intelligibility while smearing fine spectral information of the words. The filter bank spanned 80 Hz and 8000 Hz, with equal bandwidths logarithmically. For each frequency band, a Butterworth band-pass filter was created using the designfilt.m function in Matlab R2014b, with the lowest order to accommodate the specified frequency response: 25 dB down at stopbands and reduced passband ripple (less than 1 dB). The carrier within each band was generated with low-noise noise (LNN) [24], which has minimal envelope fluctuation over time, and the envelope was extracted using the Hilbert transform [25]. To eliminate energy leakage outside the designated band, the amplitude-modulated LNN was again band-pass filtered and normalized to the RMS level of original narrow-band signal prior to summation of signals of all channels. Zero phase shift was ensured by filtering the signal in both forward and reverse directions. The above series of filtering resulted in a transition region slope around 1400 dB/octave, thus reducing interference between neighboring channels.

To normalize the two audio conditions (i.e., *intact* and *vocoded*) in terms of timing and frequency profile, in the *intact* condition, speech sounds were filtered below 8 kHz using a Butterworth low-pass filter (50 dB down in the stopband and 1 dB passband ripple). Furthermore, to ensure common onset timing between *intact* and *vocoded* conditions, we implemented the following steps: (1) prior to processing, zero padding of 1000 samples (21 ms) was applied to the start and the end of the original speech signal, which also eased potential filtering edge effect; (2) subsequent to envelope replacement, sine-squared ramps were applied to the zero padded segments (phase ranging from 0 to π/2 at the beginning of the signal, and from π/2 to π at the end). Note that the zero padding edge samples would always become non-zero after filtering, due to the nature of the Infinite Impulse Response (IIR). Subsequently, the amplitude envelope of the *vocoded* signal was replaced with that of the original speech tokens through point-by-point division and multiplication. Extraction of the envelope was as follows: first, the Hilbert envelope was computed and filtered with a 15th order Butterworth low-pass filter (cutoff at 23 Hz); second, the signal was full-wave rectified and filtered again through a 10th order low-pass Butterworth filter (cutoff at 46 Hz); finally, the signal was upshifted by a 0.005 constant value to avoid discontinuities when calculating the reciprocal of a tiny value. Replacement of envelope was done to eliminate possible confounding effects of the speech envelope due to the distortion caused by vocoding. Finally, the *intact* and *vocoded* words were normalized to the same dBA level in Adobe Audition 3.0 (Adobe Systems Inc., San Jose, CA, USA).

### 2.4. Preparation of Video Stimuli

**Figure 1:**
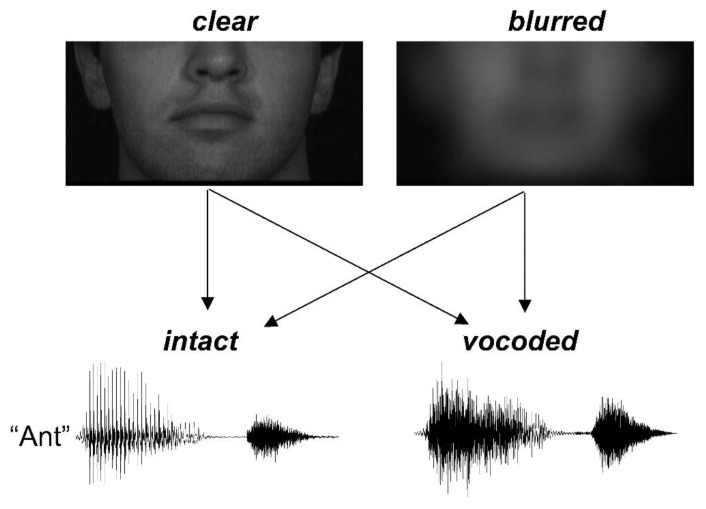
Depiction of stimulus design.

The video stimuli were manipulated using in-house Matlab code. Videos of the person’s face were initially cropped to 396 × 192 pixels size, with the mouth centered and the area above the nose excluded (Figure 1). The design included two reliability levels: *clear* and *blurred*. For the *blurred* condition, videos were filtered with a Gaussian kernel (filter size = 65 × 65, SD = 16) using fspecial.m function. The purpose of using this blurring level was largely to preserve the global temporal cues of opening and closing the mouth while smearing spatial details necessary for specific phoneme identification, such as relative movement of the tongue relative to the teeth.

### 2.5. Procedure

Individuals sat in a sound-attenuated room, 95 cm in front of a 24-inch Dell monitor. A Vizio sound bar (model S2920W-C0) was directly placed below the monitor, with one speaker vertically aligned with the center of the monitor from which a mono sound was presented. Presentation of AV stimuli were delivered by Presentation Software (v. 18.1, Neurobehavioral Systems Inc., Berkeley, CA, USA; https://www.neurobs.com/). To mimic the size of human face, the videos were rescaled to a size of 594 × 288.

The main experiment involved an auditory oddball lexical-semantic task. The participant was required to attend to the audiovisual word and press button 1 with their left hand if they thought the word belonged to the oddball category. There were 8 blocks in total, 2 for each audiovisual (2 × 2) condition. One of the two blocks was animate-dominant while the other was inanimate-dominant. For half of the participants, the four animate-dominant blocks were administered before the four inanimate-dominant blocks, with a long break in between; for the other half, the order of presentation was reversed. Block order was counterbalanced using a Latin square. The dominant/oddball ratio in each block was 90:9. Based on local oddball algorithm, the number of dominant trials between two oddball trials ranged from 5 to 15, under a triangular distribution symmetrical around 10. Among the 90 dominant trials, all 40 words in that category appeared at least twice and no more than three times. The 9 words in the oddball trials were randomly selected with no repetition. In 9 out of the 90 dominant trials, a green annulus would appear after the video was launched. In this type of trial (referred to as “probe trials” hereafter), participants were required to press “1” regardless of whether the word they heard was in the oddball category or not. These trials were later used to identify participants who did not attend to the visual stimuli and thus were excluded from further data analysis.

Each trial lasted about 5.1 s, starting with a static image—the first frame of the video, which lasted for 700–1000 ms varying across trials to jitter mouth movement onset. Time of acoustic onset was further jittered due to its naturally variable latency relative to the mouth movement onset. With the duration of acoustic signals ranging from 590 ms to 1136 ms, the participants had at least 1.8 s to respond after the end of a stimulus. The final frame of the video remained on the screen until the next trial started, leaving no gap between trials. The presented words had an average sound intensity level of 54 dBA (average of slow and fast time constant) and a peak level at 73 dBA. These values were measured using a Larson Davis SoundTrack LxT1 sound level meter, based on concatenation of thirty randomly selected audio stimuli with silent gaps removed. Responses were logged by Presentation software.

Prior to the main testing blocks, there was a training session and a practice session. In the training session, 80 words (audio and text only) were looped in a random order and participants were asked to categorize the word by pressing “3” for animate and “4” for inanimate. This served as a means of identifying any word that looked unfamiliar or ambiguous to a participant (very rare). Feedback was provided after each trial, with debriefing after the session. The practice session used the same task as the test session. Stimuli included 10 words in each category that had been selected based on feedback from a pilot study, with both audio and video degraded. From the practice phase onward, participants were encouraged to reduce eye blinks during stimulus presentation until they responded. Participants were always required to respond to the animate words by pressing “1,” although there was no real oddball in the practice session. In total, 5 out of the 20 trials were randomly selected to serve as probe trials, as described above. During the practice session, some participants realized that it was difficult to distinguish between *vocoded* word pairs like “shrew” and “shoe” or “cat” and “hat”. This was rare, but noteworthy.

### 2.6. EEG Acquisition

EEG was acquired of all subjects using 64-channel BioSemi Active Two system (10–20 Ag-AgCl electrode system, Common Mode Sense and Driven Right Leg passive electrodes as the ground). EEG was acquired at a sampling rate of 1024 Hz with no high-pass filter. The EEG signal was recorded with the DC offset and drift, with an antialiasing low-pass filter (cutoff set at 1/5 of the sample rate). EEG data were down-sampled to 256 Hz using BioSemi Decimator tool after data collection. To prevent aliasing, the Decimator uses a fifth order cascaded integrator-comb filter.

### 2.7. Data analysis

#### 2.7.1. Behavior

Response accuracy for the probe trials (72 for each person) was isolated from the analysis of other trials to assess the participant’s attention to the visual stimuli. For the remainder, besides total accuracy, each audiovisual condition was tallied respectively, collapsing across animate- and inanimate-dominant blocks. A 2 × 2 repeated measure ANOVA was conducted to see if audio and/or video degradation did substantially affect intelligibility and decision making, assuming participants did fully benefit from the training and practice sessions.

#### 2.7.2. EEG Preprocessing

EEG data were preprocessed using EEGLAB 13 [26]. For each participant, continuous data for each block were high-pass filtered at 0.5 Hz using a third order Butterworth filter, epoched from −1.5 to 2.5 s relative to the acoustic onset and re-referenced to the 64-channel global average. Datasets from different blocks were then merged. Trials were rejected if the signal exceeded ±100 µV within the time window from −100 to +690 ms in 5 prefrontal chancels (FP1, AF7, FPz, FP2, AF8) to avoid the contamination due to eye blink during the video presentation, based on the assumption that each eye blink lasts approximately 200 ms. PCA-based ICA was performed on concatenated data, resulting in 63 components, and major ICA components associated with ocular activities were removed. Bad channels, if they existed (e.g., due to bridging near the CMS/DRL electrodes), were excluded from ICA and interpolated with surrounding channels following ICA reconstruction. Trials were re-epoched from −500 to 750 ms around sound onset and baselined to the pre-stimulus prior −500 to −400 ms to avoid overlap with visually evoked activity present prior to sound onset. All trials with signals exceeding ±100 µV were rejected. Probe trials, oddball trials, and trials without a single valid response were excluded from the dataset. Event-related potentials (ERPs) were created by averaging across trials for each condition (main effects: *clear*, *blurred*, *intact*, *vocoded*; specific effects: *clear-intact*, *clear-vocoded*, *blurred-intact*, *blurred-vocoded*). The final mean numbers and standard deviations of trials per condition were as follows: *clear-intact* = 122.5 ± 21.4; *blurred-intact* = 121.5 ± 15.7; *clear-vocoded* = 119.6 ± 22.6; *blurred-vocoded* = 117.7 ± 21.2. These numbers increased for the main effect contrasts (*clear*, *blurred*, *intact*, *vocoded*).

### 2.8. Statistical Analyses

Statistical analyses of ERPs between conditions were initially conducted in Fieldtrip [27]. Given the high correlation between the data at different spatial locations (electrodes) and time points, a cluster-based statistic method was selected to alleviate the multiple comparison problem when comparing multi-dimensional data in different conditions, based on the assumption that strong neural effects tend be appear in spatiotemporally continuous clusters [27,28].

To examine the main effects of audio or visual reliability (e.g., *intact* vs. *vocoded*), the ERP waveforms of the two conditions were subjected to paired cluster-based permutation tests (CBPT). All 64 channels and sample time-points (−500 to 750 ms) were included. First, we performed two-tailed paired-samples *t* tests on the waveforms’ amplitude values of the two conditions for each channel and sample to determine univariate effects at the sample level. Only data samples with *t* values that surpassed a 0.05 probability level (two-tailed) were subsequently considered for cluster formation. Neighboring time points and channels with a univariate 0.05 *p* values were grouped together. The FieldTrip’s triangulation method was used to define neighboring channels. The final cluster-level statistics were calculated as the sum of all the t values within each sample-channel cluster. To evaluate the cluster-level significance, a nonparametric null distribution was created using a Monte Carlo approximation by repeating the above steps for 5000 random partitions (i.e., permutations) of the data, whereby the labels for the two conditions (samples and channels) were randomly shuffled. Following each permutation, the maximum of the cluster-level test statistics was used to create the null distribution. Significance was finally calculated by comparing the real cluster-level test statistics with the null distribution of maximum cluster-level statistics. Cluster-based differences between conditions were deemed significant if the cluster’s Monte Carlo *p* value was 0.05.

We visualized the CBPT results as waveforms at channel FCz (one of several channels, e.g., Fz and Cz, traditionally used to indicate auditory activity) and as *t*-value topographies distinguishing the two conditions. To obtain the t-value topographies, the *t*-values obtained by the CBPT are averaged within the cluster window of significance for each channel and then the 64 averaged *t*-values are assigned to their locations on the circle and linearly interpolated over the entire circle to reflect smooth transitions among channels. The level of t-value significance is denoted by a color map.

## 3. Results

### 3.1. Behavior

Performance (mean ± SD) was as follows: *clear-intact*, 97.6 ± 1.75%; *blurred-intact*, 97.6 ± 1.99%; *clear-vocoded*, 95.3% ± 2.4%; *blurred-vocoded*, 94.5 ± 3.25%. None of the participants reached 100% (ceiling) performance on any condition. There was an advantage of auditory reliability. Despite small percentage differences, dependent sample *t*-tests between conditions of the auditory reliability, with the same visual reliability condition, revealed that performance was significantly more robust for *intact* than *vocoded* stimuli regardless of the visual reliability condition (*p* < 0.001; Bonferroni corrected/4). Vocoding did cause difficulty in understanding some words, which was expected since some phonemes became less distinct from others after the spectral information was smeared (e.g., cat vs. hat). The high accuracy in the different conditions as well as in the probe trials indicates that the vocoding level largely preserved intelligibility. On the other hand, there were no significant differences between the visual reliability conditions (*p* > 0.3).

### 3.2. Event Related Potentials (ERPs)

We begin the ERP analyses by examining the main effects of auditory (*vocoded* vs. *intact*) and visual (*blurred* vs. *clear*) reliabilities using the cluster-based permutation test (CBPT). We then test for interactions among all conditions (*clear-intact*, *clear-vocoded*, *blurred-intact* and *blurred-vocoded*) via post-hoc analyses of variance (ANOVAs, Statistica v13, TIBCO Software Palo Alto, CA, USA). The ERP value for each condition used in the ANOVA, was based on the mean ERP amplitudes within the significant time windows of the CBPTs, averaged across a frontocentral cluster of channels (F1, Fz, F2, FC1, FCz, FC2, C1, Cz, C2).

#### 3.2.1. Auditory Reliability

**Figure 2:**
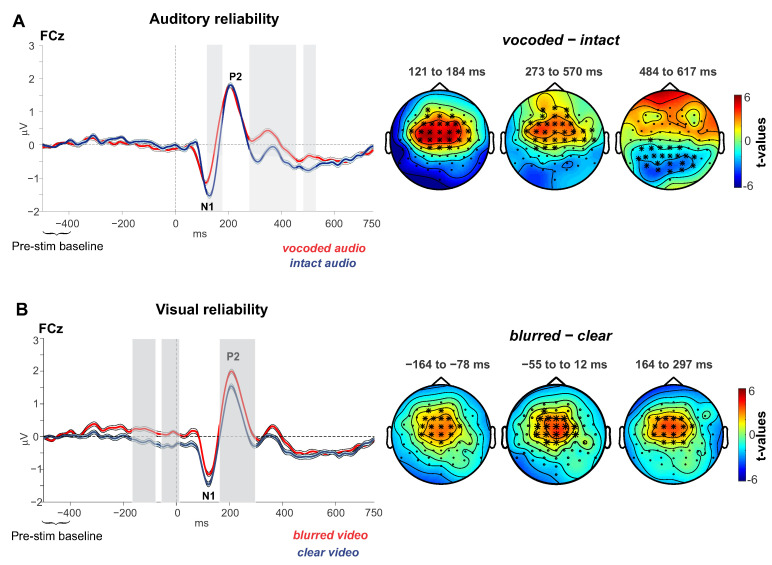
ERP waveforms and t-value topographies distinguishing Auditory reliability conditions (**A**) and visual reliability conditions (**B**). Grey shaded areas indicate significant time windows distinguishing the two conditions at channel FCz. Ribbons around the waveforms indicate within-subject standard errors. Waveforms were baselined to the −500 to −400 ms pre-acoustic onset period.

We used the CBPT to compare the *intact* and *vocoded* ERPs. Figure 2A left panel shows the ERP waveforms at channel FCz of the two conditions. Shaded areas indicate where significant differences occurred between the conditions. The right panel shows the *t*-value topographies for clusters that showed significant differences (*n* = 3 clusters). The results show that the *intact* audio stimuli evoked larger N1 ERPs (121–184 ms; *p* = 0.0004) and a larger subsequent broad negativity (273–570 ms or N450 for simplicity; *p* = 0.005) than the *vocoded* audio stimuli. The *t*-value topographies (showing positive shifts for *vocoded* minus *intact* conditions) are consistent with typical auditory topographies (frontocentral and temporooccipital) for both the N1 and the later negativity. Finally, the last cluster shows an effect (*p* = 0.004) that is largely reflected at parietooccipital cites (best observed at channels Oz and POz, not shown), whereby a sustained negative posterior shift was observed for the *vocoded* vs. *intact* stimuli. Its topography is not consistent with the earlier auditory topographies, and thus we cannot conclude that it represents the same neural generators. We do not discuss this component further.

Based on the auditory-reliability CBPT we obtained the mean ERP values within the significant time windows, averaged across the nine abovementioned frontocentral cluster of channels for the *clear-intact*, *clear-vocoded*, *blurred-intact*, and *blurred-vocoded* conditions. We subjected these values to a post hoc ANOVAs to assess for interactions. The ANOVA with the variables auditory-reliability and visual-reliability for the earlier time window (N1: 121–184 ms) revealed a main effect of auditory-reliability (*F*_(1, 24)_ = 41.3, *p* = 0.00001; η^2^ = 0.63), confirming the CBPT’s N1 effect. There was also a main effect of visual-reliability (*F*_(1, 24)_ = 6.17, *p* = 0.02; η^2^ = 0.2), which was not observed in the CBPT analysis. This is not surprising given that the post hoc ANOVA is less statistically conservative than the CBPT. Finally, there was no interaction between the variables (*F* = 0.45). A similar ANOVA for the later negativity (N450: 273–570 ms) revealed the main effects of auditory-reliability (*F*_(1, 24)_ = 21.3, *p* = 0.0001; η^2^ = 0.47), confirming the CBPT analysis, and visual-reliability (*F*_(1, 24)_ = 5.2, *p* = 0.03; η^2^ = 0.18)—not observed with the CBPT analysis. As a note, the visual reliability effect was observed in 19 out of 25 subjects. There was no interaction between the variables (*F* = 0.46).

#### 3.2.2. Visual Reliability

We used the CBPT to compare the *clear* and *blurred* ERPs. The left panel of Figure 2B shows the ERP waveforms at channel FCz of the two conditions. Shaded areas indicate where significant differences (*p* < 0.05) occurred between the conditions. The right panel shows the *t*-value topographies for clusters that showed significant differences (*n* = 3 clusters). The results show that *clear* vs. *blurred* videos evoked smaller ERPs prior to sound onset −164 to −78 ms and −54 to 12 ms; *p* < 0.05) and about 164–297 ms (P2: *p* = 0.0064) post sound onset. However, there were no cross-modal effects on the late semantic-related negativities, as we had predicted. *t*-Value topographies of all significant clusters were consistent with auditory generators (frontocentral and temporooccipital).

Based on these significant time windows, we obtained the mean ERP values at the frontocentral cluster of channels for the conditions *clear-intact, clear-vocoded, blurred-intact*, and *blurred-vocoded*, and subjected these values to a post hoc ANOVAs to test for interactions. The two ANOVAs with the variables auditory-reliability and visual-reliability for the two pre-acoustic onset periods (−164 to −78 ms and −54 to 12 ms) revealed main effects of visual-reliability (1st period: *F*_(1, 24)_ = 14.4, *p* = 0.0009; η^2^ = 0.38; 2nd period: *F*_(1, 24)_ = 16.8, *p* = 0.0004; η^2^ = 0.41) and no effects of auditory-reliability or interaction between the variables (*F* < 1.5). An ANOVA for the later period (P2: 164–297 ms) revealed main effects of auditory-reliability (*F*_(1, 24)_ = 5.7, *p* = 0.025; η^2^ = 0.19) and visual-reliability (*F*_(1, 24)_ = 18.3, *p* = 0.003; η^2^ = 0.43) with no interaction between the variables (*F* = 0.25). The P2 auditory-reliability main effect was observed in 15 out of 25, which calls into question whether this P2 effect based on the post hoc ANOVA is reliable. Nonetheless, these results again show that effects exposed in the less conservative post hoc ANOVAs, are common to both auditory-reliability and visual-reliability contrasts. Consequently, the ANOVA’s results are not taken as confirmation of significant differences, rather they are regarded as tendencies.

## 4. Discussion

We examined the neurophysiological underpinning of speech intelligibility as a function of auditory and visual reliability. We used *vocoded* (24-channel) audio words and *blurred* videos of a speaker uttering these words to assess the behavioral performance and ERPs of these conditions relative to the more reliable conditions (*intact* audios and *clear* videos). Participants performed a semantic task, whereby they identified whether the words were of animate or inanimate meaning. We hypothesized that enhanced visual reliability should result in suppression of early auditory ERPs, a process associated with enhanced AV integration efficacy [9,11,13,17], and enhanced late ERP negativities (e.g., N450), associated with enhanced semantic encoding [22]. That is, we predicted that stronger AV integration should lead to better phonemic encoding and, in turn, enhanced semantic encoding. Because the latency of the late negativity (N450) begins before 300 ms and the word stimuli were longer in duration (>500 ms), we interpret the late negativity as reflecting semantic prediction as opposed to semantic identification.

First, we found that the auditory P2 ERP, and ERPs occurring before sound onset, were suppressed for *clear* vs. *blurred* videos. This effect was not observed for the N1 ERP. The P2 has been shown to be modulated by speech-specific cross-modal inhibition, while the N1 cross-modal suppression has been associated with speech and non-speech conditions [17,29]. The findings from Stekelenburg and Vroomen [29] point to the N1 suppressive effect as being driven by temporal correspondence between the two modalities, as opposed to higher-level contextual content. However, the current data suggests that the effect is broader than a specific auditory ERP component. Based on the less conservative post hoc ANOVA, *clear* videos tended to evoke larger N1s than *blurred* ones, as compared to smaller P2 for *clear* than *blurred* conditions. In other words, the N1 and P2 shifted in one direction. This effect is reminiscent of the findings from Shatzer et al. [13], whereby *clear* videos evoked smaller auditory P1 and P2 ERPs but larger N1s for *blurred* videos. We may then acknowledge that the suppressive effect, likely instigated by the preceding visual context, is more consistent with a superimposed slow wave that begins prior to sound onset and continues past the P2 wave. The topographies of this wave suggest that it is more likely auditory in origin. If indeed AEP suppression is a consequence of a redundancy created by the preceding visual context [9,11], then we should not be surprised if suppression occurs before sound onset, and occurs independently of the early auditory ERPs (e.g., N1–P2).

Second, the cross-modal effect of visual reliability on the semantic-related N450 wave was only observed in the less conservative post hoc ANOVA—*clear* evoked larger N450 than *blurred*. Semantic prediction is reinforced by phonetic reliability [19], as demonstrated by the current auditory reliability contrasts. Furthermore, in addition to the suppressive quality, the visual modality has been shown to modify phonemic representations to emphasize the visually conveyed phonemes [7,8]. Thus, we expected a stronger N450 distinction between the two visual reliability conditions. A possible explanation for the lack of a strong visual reliability effect on the N450 may have been our stimulus choices. We used words with phonemes with strong visual-to-auditory correspondence, such as/b/and/w/, and words with phonemes with weak visual-to-auditory correspondence, such as/g/and/h/. Consequently, the inclusion of words with weak visual-to-auditory correspondence may have diluted the visual reliability effect and in turn hindered a robust emergence of semantic advantage.

Third, the cross-modal semantic prediction effect may have been weakly captured due to the high reliability of the vocoded stimuli (accuracy > 90% on average). We know from previous studies that visual context is more beneficial as acoustic degradation or noisy background increases [1,2,3,30], consistent with the principle of inverse effectiveness in multisensory integration [31,32]; but see [33]. Increased adversity in the acoustical environment may cause individuals to concentrate more on mouth movements [34,35], leading to greater engagement of the visual modality with the auditory modality. By using 24-channels vocoding, we were hoping to balance listening effort between the natural and degraded speech tokens, to rule out listening effort as a confound, while at the same time still examining neurophysiological sensitivity to semantic prediction. This may have been a shortcoming of our design.

In conclusion, the current study extends the neurophysiological cross-modal suppressive account to linguistic tasks, but also provides evidence that visual context tends to influence semantic encoding as well. Future studies addressing the current topic should consider using more degraded auditory stimuli to fully capture the semantic-related visual effect.

## Supplementary Materials

The following are available online at https://www.mdpi.com/2076-3425/10/11/810/s1, Table S1: Words used. The original data of the research are available online: https://figshare.com/articles/dataset/Role_of_visual_context_on_speech_intelligibility/13158398.
