# Supplementary material for: The Cross-Modal Suppressive Role of Visual Context on Speech Intelligibility: An ERP Study
Source: Brain Sci. 2020 Nov 2;10(11):810. doi: 10.3390/brainsci10110810 (PMC7692090; doi:10.3390/brainsci10110810)
Supplement: Supplementary file 1 [file brainsci-10-00810-s001.pdf]

Animate Words	Inanimate words
Ant	Axe
Bear	Bag
Bee	Bike
Boar	Boat
Cat	Book
Cow	Box
Crab	Bus
Crow	Car
Deer	Chair
Dog	Chalk
Eel	Comb
Fish	Couch
Fly	Cup
Fox	Desk
Frog	Door
Goat	Fork
Goose	Frame
Horse	Glass
Lamb	Hat
Lynx	Jar
Moth	Key
Owl	Kite
Pig	Lock
Prawn	Pan
Quail	Pants
Rat	Pin
Shark	Plate
Sheep	Purse
Shrew	Rail
Shrimp	Ring
Skunk	Rock
Snail	Shelf
Snake	Shirt
Squid	Spoon
Swan	Stone
Toad	String
Wasp	Tile
Whale	Truck
Wolf	Watch
Yak	Wheel

Table 1: Words used
